# Optimization of Dynamic Sit-to-Stand Trajectories to Assess Whole-Body Motion Performance of the Humanoid Robot REEM-C

**DOI:** 10.3389/frobt.2022.898696

**Published:** 2022-06-28

**Authors:** Felix Aller, Monika Harant, Katja Mombaur

**Affiliations:** ^1^ Optimization, Robotics and Biomechanics, Institute of Computer Engineering, Heidelberg University, Heidelberg, Germany; ^2^ Department of Mathematics for the Digital Factory, Fraunhofer Institute for Industrial Mathematics, Kaiserslautern, Germany; ^3^ CERC Human-Centred Robotics and Machine Intelligence, University of Waterloo, Waterloo, ON, Canada

**Keywords:** humanoid robots, whole-body control, legged robots, optimization, optimal control, benchmarking

## Abstract

To enable the application of humanoid robots outside of laboratory environments, the biped must meet certain requirements. These include, in particular, coping with dynamic motions such as climbing stairs or ramps or walking over irregular terrain. Sit-to-stand transitions also belong to this category. In addition to their actual application such as getting out of vehicles or standing up after sitting, for example, at a table, these motions also provide benefits in terms of performance assessment. Therefore, they have long been used as a sports medical and geriatric assessment for humans. Here, we develop optimized sit-to-stand trajectories using optimal control, which are characterized by their dynamic and humanlike nature. We implement these motions on the humanoid robot REEM-C. Based on the obtained sensor data, we present a unified benchmarking procedure based on two different experimental protocols. These protocols are characterized by their increasing level of difficulty for quantifying different aspects of lower limb performance. We report performance results obtained by REEM-C using two categories of indicators: primary, scenario-specific indicators that assess overall performance (chair height and ankle-to-chair distance) and subsidiary, general indicators that further describe performance. The latter provide a more detailed analysis of the applied motion and are based on metrics such as the angular momentum, zero moment point, capture point, or foot placement estimator. In the process, we identify performance deficiencies of the robot based on the collected data. Thus, this work is an important step toward a unified quantification of bipedal performance in the execution of humanlike and dynamically demanding motions.

## 1 Introduction

Many of the whole-body motions we perform as humans in everyday life appear simple at first glance. Only as we grow older, we become aware of their complexity. The decrease in our physical abilities that accompanies age leads to more conservative behavior when performing physically challenging motion tasks. In itself, this speaks for their level of difficulty: dynamic motions like running, climbing stairs, jumping, or standing up from a chair Sloot et al. (2020) require proper leg muscle output, coordination, mobility, and the control of balance at the same time Millor et al. (2014). Implementing such motion sequences on a humanoid robot is equally challenging, as it also demands the aforementioned aspects from the underlying mechanical system Gu and Ballard (2006). While sitting, the center of mass (CoM) initially lies within a support area which is defined by the convex hull of the chair, that is, the chair legs, and the robotic feet. The difficulty is further increased by the possibility of a statically unstable configuration after breaking the sitting contact. Generally, the motivation to implement sit-to-stand (StS) trajectories on a humanoid robot is twofold:

• The development of complex motion patterns which correspond to natural human motion.
• The evaluation of the robot based on challenging dynamic motion patterns.

Both reasons require contributions of the whole body, are equally important, and are also partly dependent on each other.

The former refers to operating in a built-for-human environment. Efforts must be made to ensure that robots will one day be able to interact with objects intended for humans, such as chairs Kemp et al. (2007). This is conceivable both in domestic applications, where a robot sits to save energy while performing sole manipulation tasks, or even in industrial or disaster scenarios, where robots get out of a vehicle that may transport them to, for example, an emergency site Bouyarmane et al. (2012).

The latter includes preparation of the robot for the former domains and scenarios. Many dynamic motions require the robot to be mechanically capable of the task at hand. To ensure functionality, quantitative and unified benchmarking based on various metrics is a favorable approach. It requires practical, simplified scenarios and experimental protocols that simulate real-world challenges and can be replicated in a number of different laboratories Torricelli et al. (2015).

Some standardized experimental setups and protocols are already in development to assess the robustness of the robot to external disturbances Lippi et al. (2019) or the stability at different step sizes during bipedal locomotion Aller et al. (2021b). To the best of our knowledge, no benchmark has yet been presented to quantify the StS capabilities of a humanoid robot. This fact is particularly interesting because StS as an exercise has long been an essential component of whole-body performance analysis in geriatric and sports medical assessments Podsiadlo and Richardson (1991), Bohannon (1995), Jones et al. (1999). A reason for the lack of StS benchmarking in robotics is the challenge of transferring such dynamic motions to a mechanical system. Mistry et al. (2010) used motion capture recorded StS motions from human subjects and mapped the marker-data trajectory to the Carnegie Mellon/Sarcos humanoid robot using inverse kinematics. Pchelkin et al. (2010) also used analyzed human motion as a basis from which various constraints are initially derived. Starting from the angle at the ankle, the other joint angles of the knee and hip are derived using a cubic polynomial. Other classical approaches as presented by Gongor et al. (2017) control the center of pressure (CoP) and maintain it close to a stable region by shifting the robot’s upper body, resulting in a statically stable and more conservative motion. For more natural and biologically inspired motion generation that also exploits the full dynamics of the robotic system, the use of optimization, for example, an optimal control approach based on the robot’s whole-body model, is favorable. Optimal control is a promising tool in motion generation as the motions are more feasible with respect to all constraints within the model and the motion task Hu and Mombaur (2018). In the past, optimization has already been successfully used to realize demanding motions on robots such as HRP-2 Koch et al. (2012a), Koch et al. (2012b), Lengagne et al. (2013), Koch et al. (2014).

In this work we present quantitative benchmarking protocols to assess the performance of the whole body by assessment of performance indicators (PIs) based on a selection of metrics from the literature. The protocols are based on different StS trajectories that are generated by means of optimal control and carried out on the humanoid robot REEM-C. The main contributions of this study consist of the following:

• The formulation of a StS benchmark. This includes the definition of two benchmarking protocols, the development of an instrumented chair for data collection, and the standardized evaluation of experimental data based on predefined performance indicators.
• The generation of dynamically demanding motions using optimal control implemented on the humanoid robot REEM-C reaching its maximum capability. The calculations, unlike some of the previously mentioned approaches, do not require experimental data from humans.
• The execution of the StS benchmark for REEM-C based on the generated motions and the provision of experimental data and results in a uniform data format.

The carried out work took place within the European project EUROBENCH (https://eurobench.github.io). The project is dedicated to the development of testing facilities for exoskeleton and humanoid robot benchmarking on the basis of a unified testing environment. For this reason, the contributions are intended to have the highest possible degree of standardization. This enables their application in several laboratory environments and provides a comprehensive framework for the comparability of research results.

## 2 Benchmarking Scheme

Benchmarking in robotics is mostly carried out by means of competitions and challenges, in which the performance of systems is evaluated on the basis of whether they pass or fail a higher-level task. A performance analysis of the subsystems is not provided in this particular case. To analyze systems in a comprehensible and unified way, a methodological approach is needed. In this section, we present the experimental setup of the StS benchmarking performed on the REEM-C robot and its integration into a unified benchmarking environment.

### 2.1 Unified Benchmarking

**FIGURE 1:**
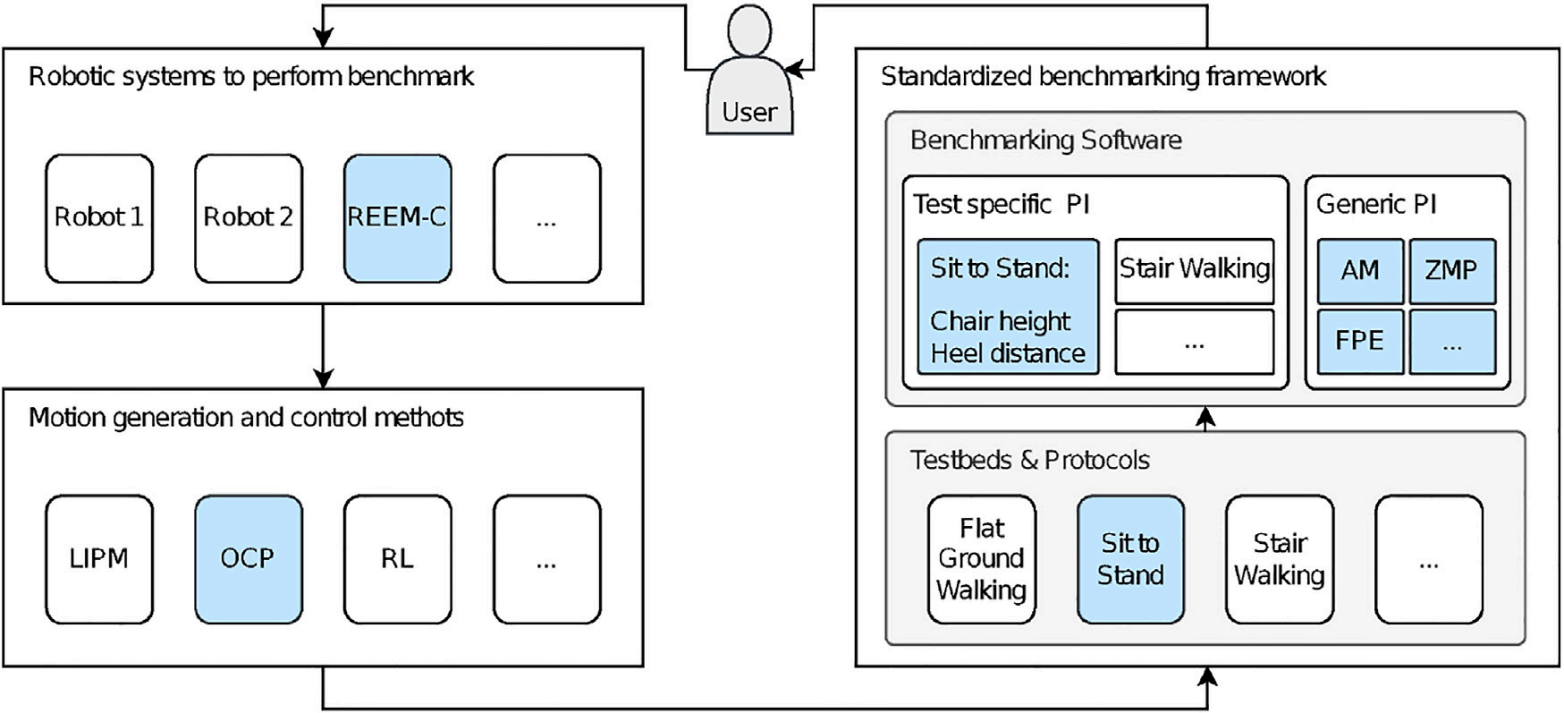
Application of a benchmarking experiment using the EUROBENCH framework: A user decides to perform experiments with a robot. A variety of different robots and associated control algorithms, for example, based on the linear inverted pendulum (LIPM), optimal control problems (OCP), or reinforcement learning (RL), are conceivable. It does not matter on what basis the motions are generated, as long as the recorded data comply with the input data format. Additionally, all robots are applicable as long as the underlying model file of the robot follows the *de facto* URDF standard. The experiments to be performed are carried out using one of the available protocols within a defined test environment. The obtained data are processed using the benchmarking software. PIs are distinguished based on two different categories. Testbed-specific PIs are calculated only with respect to the protocol and testbed used (e.g., step length or stair or seat height). Generic PIs are calculated for a variety of different protocols. The particular application presented in this work is highlighted in blue.

**FIGURE 2:**
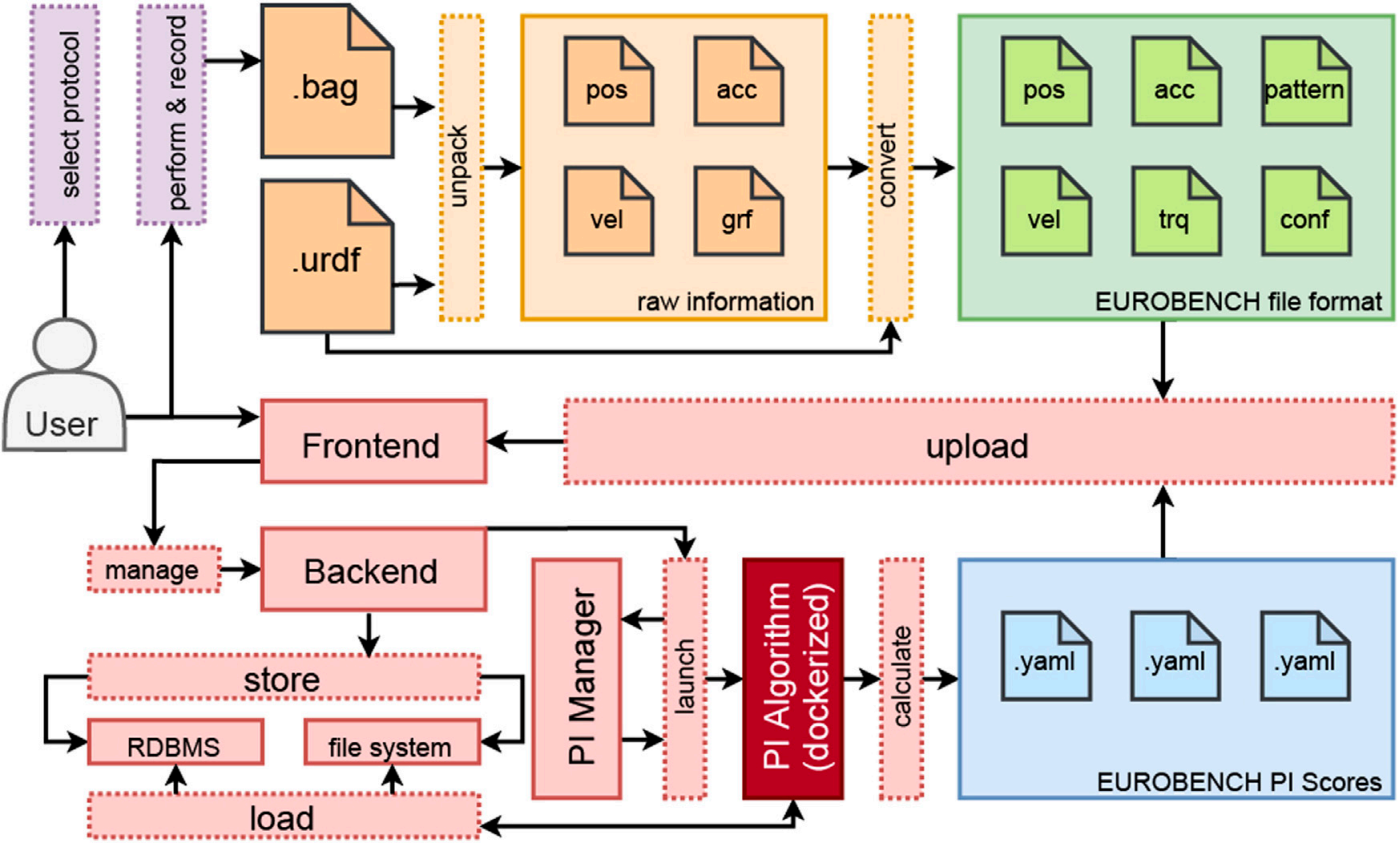
Workflow within the EUROBENCH framework: The user selects a standardized protocol from a set of predefined protocols, performs the experiment as specified, and records the experimental data. (orange) The type of data acquisition, that is, the sensors used and the data conversion, is robot and laboratory dependent and completely interchangeable. After the required preprocessing of the data into the EUROBENCH input file format, it serves as the basis for the calculation of PIs (green and red) The data used as a basis for benchmarking are provided by the user *via* the front-end of the benchmarking framework and stored in the database by the back-end. The program to calculate the PI associated with the protocol (dark red) is invoked by the PI Manager. It is executed in a Docker environment that is operating in a system-independent manner. In this work, we are focusing on the StS protocol and its associated PIs. These are output *via* .yaml files (blue) and made available to the user *via* the front-end.

Unified benchmarking is necessary to make research results from different institutions, which have been conducted under laboratory-specific conditions, inherently comparable. We define unified benchmarking as a process in which different robots with different control architectures are assessed consistently with standardized test cases of various test scenarios and the evaluation is based on standardized methods and a uniform data format (Figure 1). The three key factors that emerge from this definition are systematically applied within the deployed benchmarking procedure (Figure 2):

1) A generalized protocol to describe the experiment.
2) A data format that stores the origin of data from various sources in a common, consistent, uniform, and flexible way.
3) A piece of software that evaluates subjects, in this case, robots, by acting on the basis of this format and calculating the respective indicators to determine performance.

Protocols, as described in 1. clearly indicate what equipment is needed, what the individual steps of the test and the termination conditions are, and how individual runs differ from each other. Only if the protocol is strictly followed, the test results are subsequently comparable with each other. It does not matter in which way the robot’s motions are generated to perform the motion sequence described by the protocol. Moreover, it is desirable to test a variety of different approaches to motion generation in order to compare their results based on the same protocol. In this work, an optimal control approach to motion generation was chosen (see Section 3). We expect this to produce the most humanlike and complex motions, which can serve as additional qualitative reference datasets for subsequent runs.

The data format (2.) serves as the basis for the calculation of PIs. It must provide all necessary data but also be kept basic to be adapted by different laboratories. We rely on a collection of time-series.csv files and robot descriptive files. These files form a standard file format and are generated by different preprocessing measures (see Section 2.5). We distinguish between a position, velocity, and acceleration file, force torque files for measured ground contact forces, and a joint torque file. The robot-describing files include the robot model file in the *de-facto*-standard unified robot description format (URDF) and a robot.yaml file, which contains additional information about the robot (e.g., rotation order of the baselink, segments exposed to external contact, and position of the foot sole relative to the origin of the foot segment). Based on these files, a variety of performance data from different robots can be analyzed using the framework.

The framework acts on the basis of these unified data (3.). To make the benchmark applicable to different laboratories and to make its use more appealing, it is important to keep the barrier to adoption to a minimum. This includes the fact that the program can run on different operating systems and is based on license-free and freely available software. This software product and the interaction of the individual components is one of the major contributions of the EUROBENCH project. The benchmark for StS performance measurement presented in this study integrates as an add-on into the system landscape of the EUROBENCH benchmarking framework. The latter is available as part of the project. The general system architecture is in line with industry standards: A web front-end receives the user’s data files. The back-end stores the .csv files on a file server and links them to the experiment using the meta information stored in an SQL database. A software layer called PI Manager handles the calculation of PIs. The required data for the respective protocol is determined using the SQL database and is made available by the file server. A Docker container with the required components is created and the calculation of the PI is performed. The results are made available *via* the front-end. A complete on-premise solution in the respective laboratory without the system landscape provided by the project can also be executed locally using the respective Docker container. This facilitates a benchmarking solution for each protocol based on prior data pre-processing and customization of the robot configuration and associated robot model. All mentioned components are considered in the underlying work according to the specifications of the EUROBENCH project.

### 2.2 Sit-to-Stand Protocol

**FIGURE 3:**
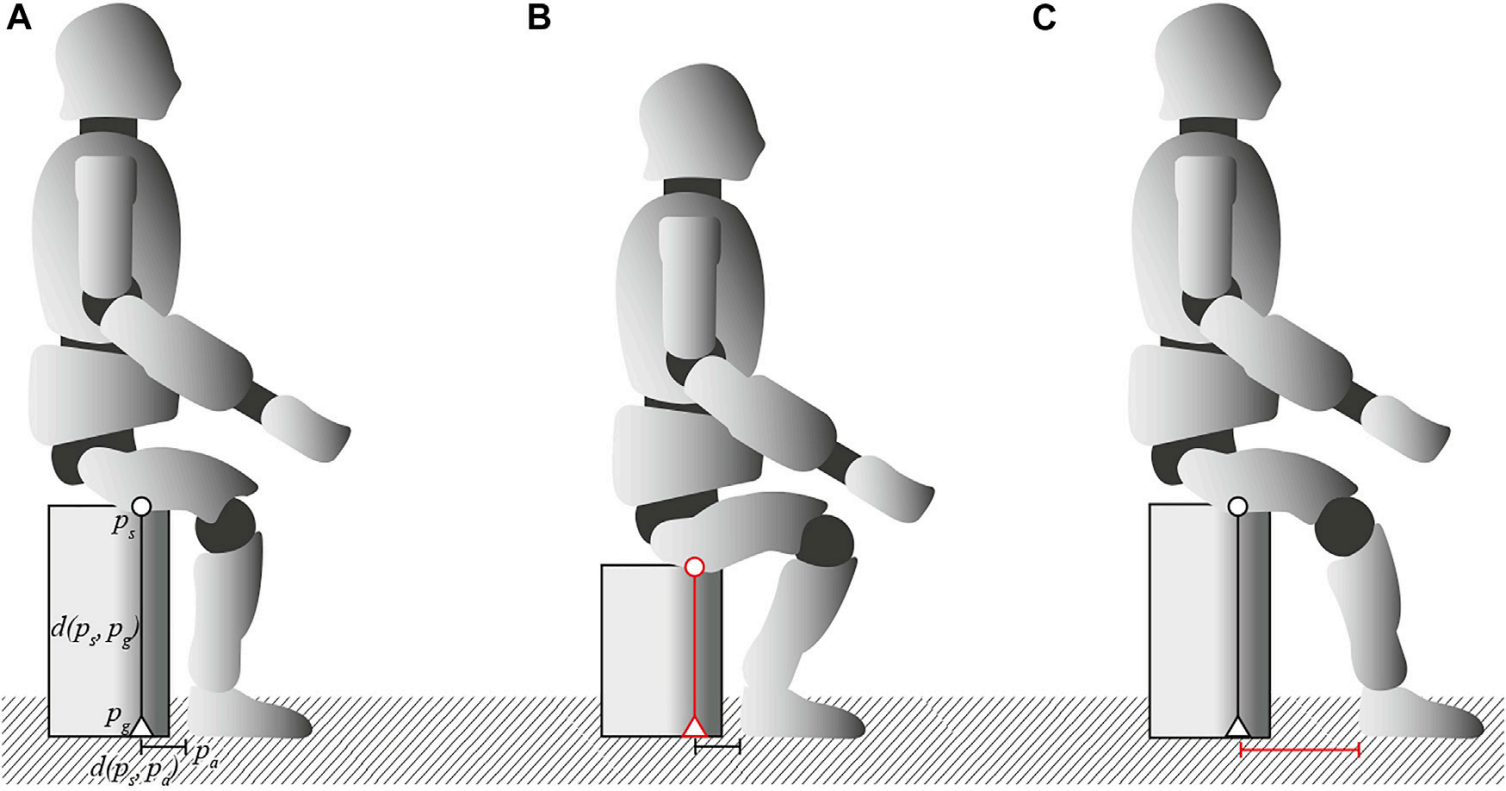
Three different sitting configurations. **(A)** is the default sitting position at 120% of the knee height with sitting contact *p*_*s*_, ground-projected sitting contact *p*_*g*_ and ankle position *p*_*a*_. The distances between *p*_*s*_ and *p*_*g*_, *p*_*g*_, and *p*_*a*_ are denoted as *d* (*p*_*s*_, *p*_*g*_) and *d* (*p*_*g*_, *p*_*a*_), respectively. **(B)** corresponds to the first protocol with *d* (*p*_*s*_, *p*_*g*_) incrementally decreasing (indicated in red) and therefore putting more stress to the knee joints, focusing on a higher vertical displacement of the CoM. **(C)** corresponds to the second protocol with *d* (*p*_*s*_, *p*_*a*_) incrementally increasing (indicated in red), requiring a horizontal and vertical shift of the CoM putting more stress onto the hip joints.

In previous research, we focused on quantifying stability during flat-ground walking Aller et al. (2021b). The robot was able to perform bipedal locomotion with step lengths of up to 40% of its total leg length without losing balance. The locomotion pattern was based on the Three-Dimensional Linear Inverted Pendulum Model (3D-LIPM) Kajita et al. (2002), which is common for bipedal robots. This type of control results in a motion keeping the center of mass (CoM) fixed at a predefined CoM height while shifting it along the lateral and longitudinal axes, which is not very demanding for the underlying motors. The extent of stress in a vertical CoM displacement only comes into play when dealing with height differences on stairs, ramps, or, in this case, by the impulsive lifting from a sitting position. Therefore, StS is very suitable as benchmark to quantify key capabilities of the robot such as its speed, strength, precision, and balance capabilities in a static experimental setup. The scenario is described by the position of sitting contact *p*_*s*_ relative to the coordinate system of the robot’s thigh segment, the sitting contact projected onto the ground *p*_*g*_, and the center of the ankle *p*_*a*_ relative to *p*_*g*_. The distance between *p*_*s*_ and *p*_*g*_ and thus the chair height is denoted as *d* (*p*_*s*_, *p*_*g*_) and *d* (*p*_*g*_, *p*_*a*_) describes the distance between *p*_*g*_ and *p*_*a*_ (Figure 3A). The primary objective of the robot is to change from a sitting to a standing configuration, that is, to stand up from a chair. For this purpose, two different protocols of increasing difficulty, *P*1 and *P*2, are applied:

• *P*1: Incremental decrease in *d* (*p*_*s*_, *p*_*g*_) to increase the vertical displacement of the CoM (Figure 3B).
• *P*2: Incremental increase in *d* (*p*_*g*_, *p*_*a*_) to increase the horizontal displacement of the CoM (Figure 3C).

We define each sitting position as a configuration where, without the appropriate sitting contact, the sitting configuration would be statically unstable and cause the robot to tip backward should the chair be removed. To prevent an actual fall, the robot is secured by means of a patient lifter. For each trial with different experimental parameters, three repetitions were performed. After every three repetitions, the difficulty was gradually increased until the robot could no longer execute the requested motion. The increase in difficulty is normalized according to the knee height of the robot, that is, the distance from the ground to the center of the knee joint while standing in null (upright) posture. An elevated position above knee height with immediate ankle distance was selected as the initial configuration for both protocols, resulting in a total of four experiments each for the first and the second protocol (Table 1).

**TABLE 1:** Experimental parameters for both protocols. Protocol 1 with decreasing chair height *d* (*p*_*s*_, *p*_*g*_) normalized according to the robot’s knee height, that is, the height from the center of the knee joint to the ground, while standing straight and constant ankle distance *d* (*p*_*g*_, *p*_*a*_). Protocol 2 with increasing ankle distance *d* (*p*_*g*_, *p*_*a*_) normalized according to the knee height and constant chair height *d* (*p*_*s*_, *p*_*g*_). For both protocols, the duration in seconds of the motion for each trial and the of the individual phases T1 and T2 (T1 before breaking contact and T2 after breaking contact) (see Section 3) is reported. Please note that *P*1 120% and *P*2 25% are the same experiments and are listed twice for the following evaluations for the reason of completeness and comparability.

Protocol 1—chair height decrease			
In % of knee height	d (p_s_, p_g_) (cm)	d (p_g_, p_h_) (cm)	Duration (T1, T2) (s)
120	49.8	10.4	1.54 (0.35, 1.19)
115	47.7	10.4	1.59 (1.20, 0.39)
110	45.7	10.4	1.63 (1.22, 0.41)
105	43.6	10.4	2.23 (1.28, 0.95)
100	41.5	10.4	2.21 (0.90, 1.31)
**Protocol 2—ankle distance increase**			
**In % of knee height**	**d (p_s_, p_g_) (cm)**	**d (p_g_, p_h_) (cm)**	**Duration (T1, T2) (s)**
25	49.8	10.4	1.54 (0.35, 1.19)
30	49.8	12.5	1.66 (0.41, 1.25)
35	49.8	14.5	1.78 (0.52, 1.26)
40	49.8	16.6	1.87 (0.40, 1.47)

### 2.3 Robot

**FIGURE 4:**
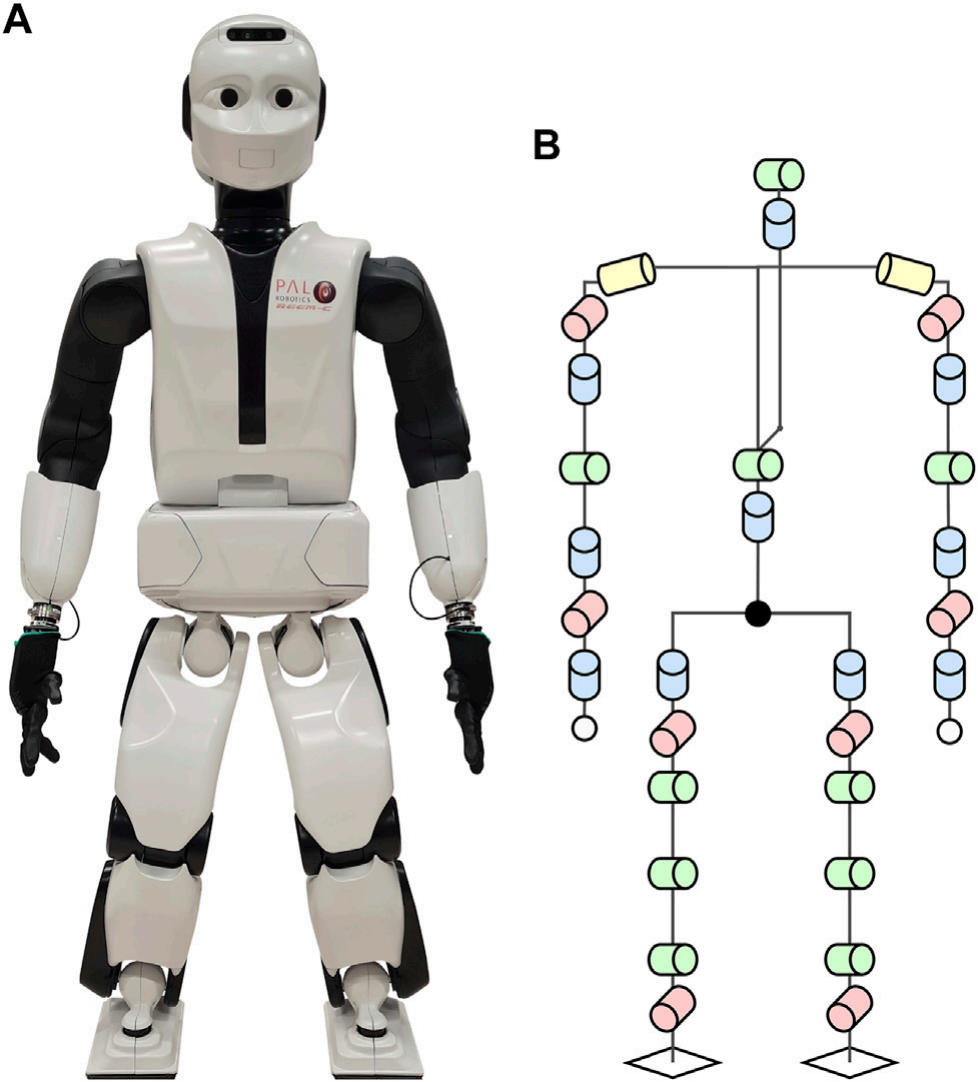
Humanoid robot REEM-C manufactured and distributed by PAL Robotics, Barcelona, Spain. **(A)** displays a frontal view of the actual robot while standing, with a total height of 1.64 m at 77.5 kg. **(B)** depicts the 30 degrees of freedom and the kinematic chain of the robot (not considering the hands). In the OCP, a model consisting of the green and yellow joints was used, with the legs and arms lumped together. The remaining joints (indicated by blue and red) were fixed.

**FIGURE 5:**
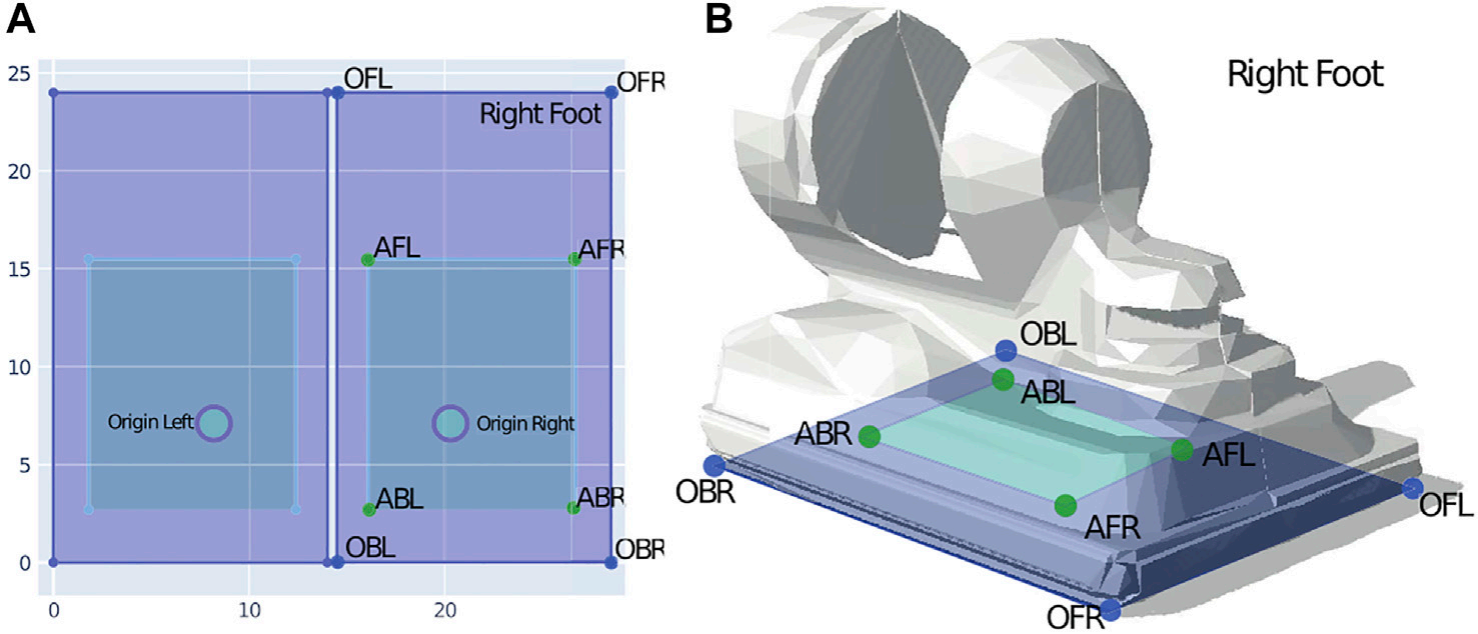
Base of support (BoS) of the robot feet. The right foot (outer rectangle) is labeled OFL—Original Front Left, OFR—Original Front Right, OBL—Original Back Left, and OBR—Original Back Right. The inner rectangle is labeled in an identical manner, with A as an abbreviation for Actual (BoS). **(A)** shows the dimensions of both feet in blue, with a width of 14 cm (OFL-OFR) and a length of 21 cm (OBR—OFR). The blue area describes the convex hull of each foot which is equal to the foot contact surface. The origin of the left and right foot is shown as a circle. The actual BoS is depicted as a green inner rectangle and is significantly smaller than the convex hull, with a width of 10.6 cm (AFL—AFR) and a length of 15.5 cm (ABR—AFR). The BoS was determined by experiments in which the robot was tilted while in null pose during single support (on one leg) and double support (on both legs) along the lateral and longitudinal axis until the robot tipped over. The labels correspond to **(B)**, which shows the right foot of REEM-C and relates the dimensions of both the actual BoS and the contact area, that is, the convex hull of the foot.

Experiments were performed with the adult-sized humanoid robot REEM-C (PAL Robotics, Barcelona, Spain) (Figure 4A) with 30 DoF (not considering both hands) (Figure 4B). REEM-C internally runs on the Robot Operating System (ROS) and utilizes the ros_control framework. The robot weighs 77.5 kg with the origin of the kinematic chain, that is, base link, at a height of 85.9 cm in null pose. The knee height of REEM-C is measured at a height of 41.5 cm above the floor. Each increase in difficulty for both protocols results in a decrease in *d* (*p*_*s*_, *p*_*g*_) or an increase in *d* (*p*_*g*_, *p*_*a*_) by 5% of the aforementioned knee height for the first or second protocol, respectively. Many of the PIs presented in a subsequent section (see Section 2.6) are based on the distance to the nearest edge of the robot’s base of support (BoS). The BoS is located inside a convex hull which is spanned by the contact surfaces of both feet when the robot is standing. In the case of REEM-C, the convex hull of the feet does not correspond to the actual BoS while standing (Figure 5A). While a foot is about 21 cm long and 14 cm wide, we determine the BoS over a range of 15.5 cm length by 10.6 cm width (Figure 5B). This significantly smaller BoS is determined based on tipping over experiments. The subsequently described PIs are all determined according to the actual smaller BoS and its edges.

### 2.4 Testbed

**FIGURE 6:**
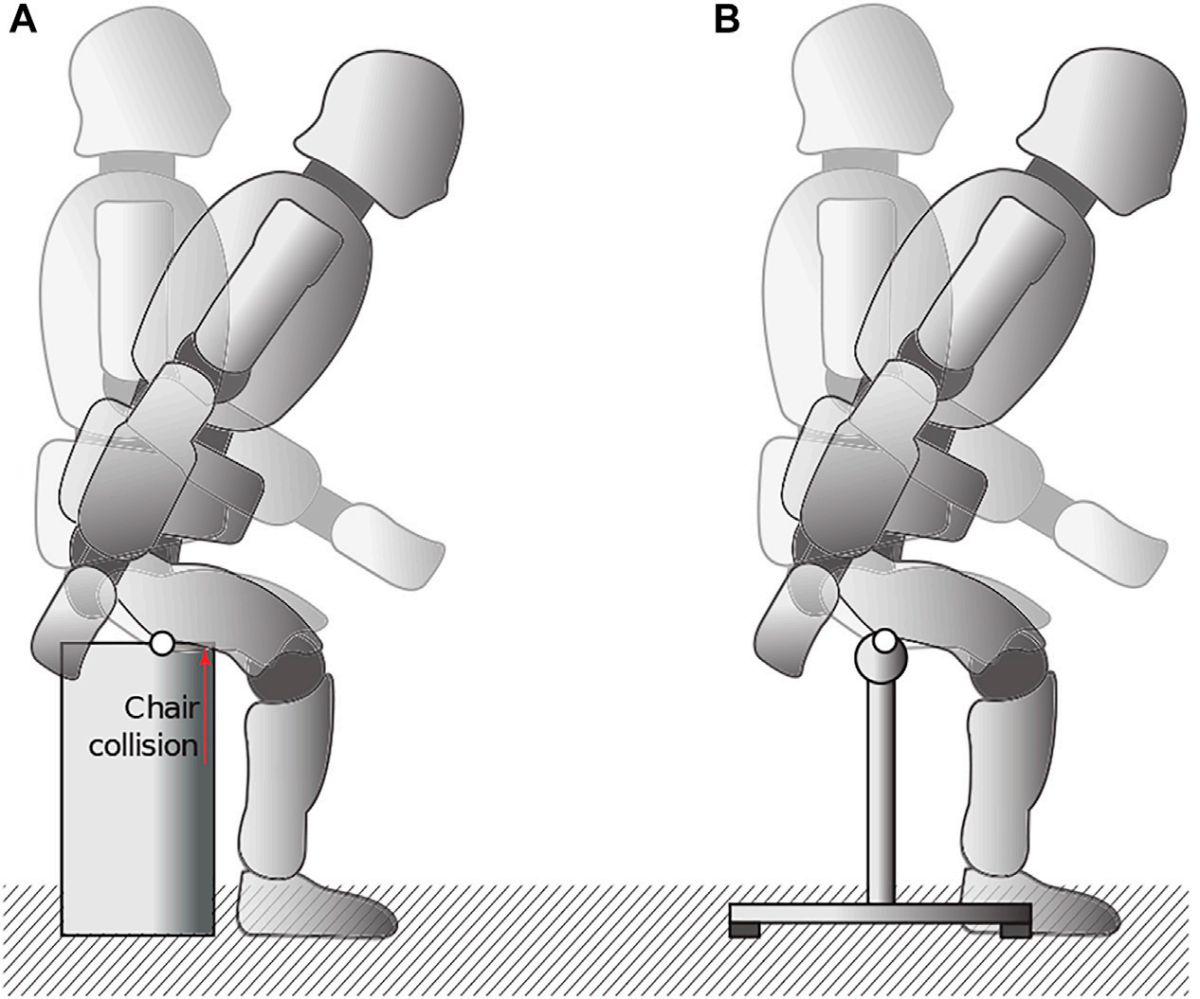
Two different sitting scenarios for a humanoid while sitting on his thigh: **(A)** with a common, flat contact surface that results in unpredictable collisions due to rotation of the rigid thigh segments and **(B)** with a cylindrical contact surface in which the thigh segment can rotate around the sitting contact, preventing additional collision.

A variety of different chairs were tested for the experimental setup. In addition to a stable chair whose seat does not yield to the impulse when contact is broken, attention was also paid to the measurement of the sitting contact force. Attempts to attach a chair to a ground-embedded force plate failed due to the lack of mounting options. The utilization of a commercial chair has been excluded not only because of the chair’s overall compliance but mainly because of the usual flat seat. Unlike humans, REEM-C has rigid multibodies and no soft tissues. Unintentional and unpredictable collisions occur during the stand-up motion due to the accompanying rotation of the thigh (Figure 6). To meet all the requirements, a custom chair was manufactured.

**FIGURE 7:**
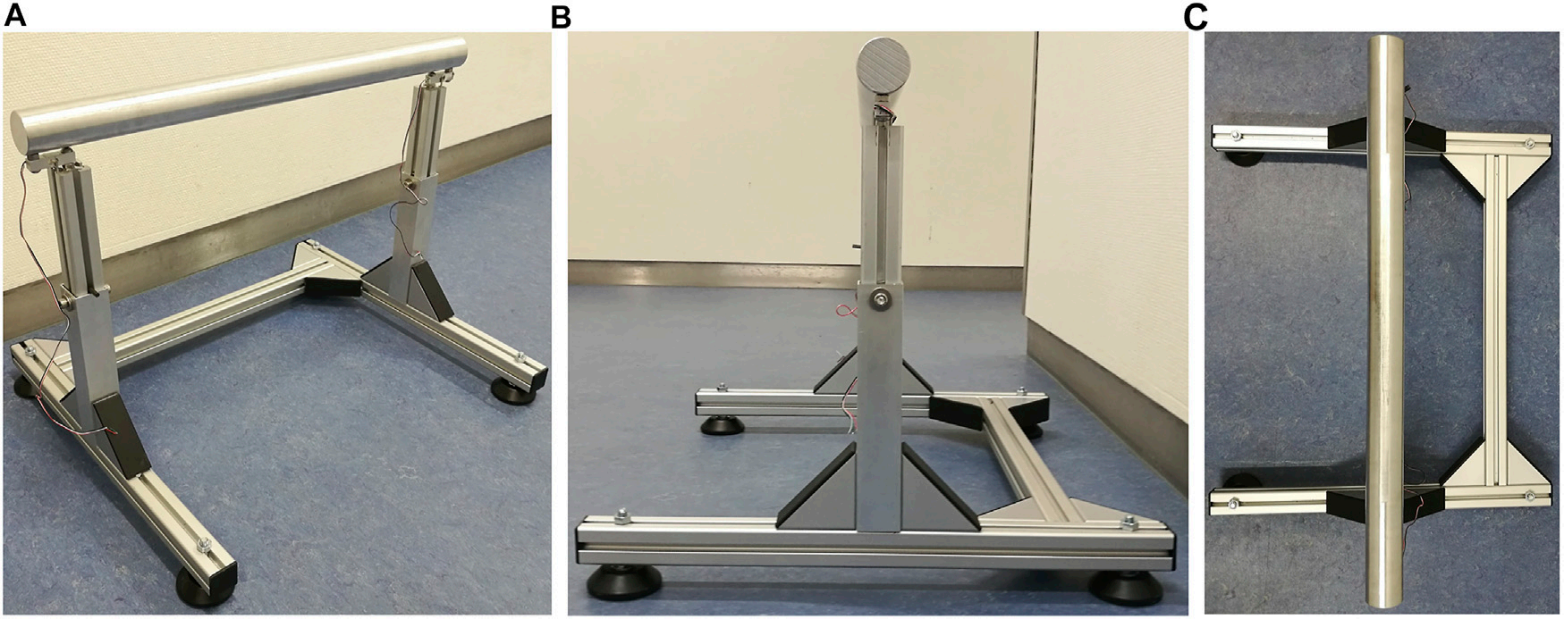
Instrumented chair with a round, 5-cm-thick aluminum alloy bar right above the center of the frame. **(A)** Frontal view of the chair with the force sensors mounted directly under the beam. **(B)** Side view of showing the height-adjustable aluminum profile. **(C)** Top view showing the leg space to the front (left side).

We developed an instrumented chair with force sensors on a horizontally aligned cylindrical beam as the contact surface, which due to its properties, always results in a defined contact point (Figures 7A–C). To measure the applied vertical force, two strain gauge load cells are utilized, which measure the force directly below the aluminum beam at its left and right attachment points. The height of the sitting contact is freely adjustable in a range between 35 and 70 cm above the floor. It is designed in such a way that the chair does not have chair legs that hinder the robot’s freedom of movement. It is also possible to place the robot feet directly below the sitting contact. The chair has a maximum load capacity of approximately 100 kg and the applied vertical force is measured at 80 Hz and transferred to ROS using one and the same wall-time, as is the case with REEM-C.

### 2.5 Data Collection

The main source for experimental data is the sensory information of REEM-C. Their quality and accuracy in comparison to reference systems have already been established in previous work Aller et al. (2021b). Apart from the measured data of the chair, no other measurement device is used for data collection. The experimental data are stored in real time into bags, the standard ROS format to store serialized sensor data. The ROS-topic information from the bags is converted into several pre-processed .csv files. The pre-processed datasets are converted to the comprehensive and unified input data format (https://eurobench.github.io/sofware_documentation/latest/data_format.html) defined within the EUROBENCH project.

As REEM-C does not provide all required data, we generate the missing information in the process of pre-processing. This includes information from the global orientation and translation of the base link calculated by means of inverse kinematics assuming a flat foot–floor contact and the use of robot internal IMU-data. Velocity and acceleration of the base link are calculated by first- and second-order derivatives of a cubic smoothing spline fit to the aforementioned data (see Section 6). Joint acceleration is determined by the same means. Joint torques are calculated employing inverse dynamics based on data obtained from the force/torque sensors in the robot’s feet and an updated version of the robotic model Aller et al. (2021a). The calculation of PIs to quantify the robotic performance is based on the calculation of metrics by means of the open-source Rigid Body Dynamics Library (RBDL) Felis (2017).

### 2.6 Applied Performance Indicators

In order to quantify the various key capabilities of the robot, these are assigned to different categories. In addition to capabilities such as perception, manipulation, interaction, configurability, decisional autonomy, and cognitive ability, these also include the capabilities addressed within the presented benchmark such as motion ability, adaptability, and dependability Robotics (2016). They all have subordinate task areas that are analyzed in this work and are defined as PIs which are again divided into generic and scenario-specific PIs. Scenario-specific PIs, which are only based on comparisons within the same scenario, as in this case with StS transitions, are determined from the category of adaptability: different chair heights and foot distances, dependability: repetition of one and the same trial with the same results, and motion ability: sit-to-stand duration and motor strength to perform the required task. Generic PIs can be applied to a variety of scenarios. We rely on PIs based off the metrics presented below that analyze motion ability or, more precisely, strength, precision, stability, and human-likeness with respect to the motion performed.

• Angular Momentum (AM): When evaluating human motion, the overall angular momentum around the CoM is often considered. Whereas many of the point-based metrics rely mostly on a simplified model of the robot, the AM takes into account all the properties of the individual segments in the rigid multibody system. These include all inertial properties and angular and relative translational motions. Humans attempt to minimize the AM to near zero for all axes during locomotion. In previous work, we already successfully applied the AM as a metric for stability boundaries and defined stability regions for the REEM-C robot Aller et al. (2021b). The AM vector is obtained by first solving the following linear system Essén (1993):

$$J_{C}\omega =H_{C}$$ (1)

with *J*_*C*_, *H*_*C*_ being the moment of inertia and AM about the CoM and ‖*ω*‖_2_ being the angular speed of the robot as a multibody system. *J*_*C*_ is then given by the following:

$$J_{C}=\overset{n}{\underset{i=0}{\sum }}J_{i}+m_{i}\left(I\left(c_{i}^{T}c_{i}\right)-\left(c_{i}c_{i}^{T}\right)\right)$$ (2)

where *n* is the number of bodies of the multibody system, *c*_*i*_ is the distance vector of the CoM to body *i*, the inertia matrix *J*_*i*_ of body *i* at the corresponding CoM and the mass *m*_*i*_ of the corresponding body. The AM is calculated by the following:

$$H_{C}=\overset{n}{\underset{i=0}{\sum }}J_{i}\omega_{i}+c_{i}\times \left(m_{i}v_{i}^{c}\right)$$ (3)

with *ω*_*i*_ as angular velocity of body *i* and $v_{i}^{c}$ as velocity of body *i* with respect to the CoM of the robotic system. For an StS motion, special attention is paid to the AM acting about the lateral axis. We normalize the AM about all axes, *AM*_*x*_, *AM*_*y*_, and *AM*_*z*_, with *ml* ^2^ to make the results comparable across robots and our previous research with *m* being the mass and *l* the leg length from the sole to the freeflyer while the robot is standing upright (see Section 2.3).

• Zero Moment Point (ZMP): The ZMP is a point-based metric and indicates the point on the ground at which the robot’s tipping moment is zero due to gravity and inertial forces. It originates in the equations of motions, which can be written as follows:

$$ma_{C}=mg+f^{c}$$ (4)

$$\dot{H_{O}}=\vec{OC}\times mg+\tau_{G}^{c}$$ (5)

where *m* is the total mass of the multibody system, *C* the location of the CoM, *a*_*C*_ the CoM acceleration, and ${\dot{H}}_{O}$ the rate of change of the AM at a given point *O*. The gravity vector is defined as *g* and *G* defines the center of gravity of the system. The net contact wrench which is the sum of all contact wrenches exerted onto the robots is calculated by the following:

$$w_{C}^{c}=\left[\tau_{C}^{c}\;f^{c}\right]$$ (6)

The gravito-internal wrench of the robot depends on its own acceleration and can be defined as follows:

$$f^{gi}=m\left(g-a_{C}\right)$$ (7)

$$\tau_{O}^{gi}=\vec{OC}\times mg-{\dot{H}}_{O}$$ (8)

Equations 7 and 8 demonstrate the dynamic balance of the multibody system in case the inertial, contact, and gravitational forces are strictly opposite. The ZMP can be calculated by means of the following:

$$p_{z}=\frac{n\times \tau_{O}^{gi}}{f^{gi}\cdot n}$$ (9)

where *n* denotes the normal vector to the ground surface. Should the ZMP reach and exceed the edge of the BoS, a flat ground contact can no longer be maintained and the multibody system will rotate at the ZMP about the corresponding edge of the BoS. The quality of the calculated ZMP is highly model dependent, as it takes into account the dynamic and kinematic properties of the individual body segments within the model. In previous publications, we have relied on the calculation of the CoP using the force torque measurements of the robot, since the CoP and ZMP correlate Sardain and Bessonnet (2004). Due to a model identification of REEM-C performed in advance, the new version of the model with updated model parameters can be used for calculation. Because of the measurement errors caused by the oscillation of the robot while standing up, the ZMP yields more accurate information and provides more meaningful results. Therefore, the calculated ZMP from the model data and motion dynamics is preferable to the CoP based exclusively on the measured values of the force-torque sensors in both feet. We expect the use of the ZMP to provide information on the static stability of the system. It should reside within the BoS during the motion. We investigate how closely the ZMP approaches the next edge of the BoS with increasing difficulty and, accordingly, how close the robot is to tipping over.

• Capture Point (CP): The CP defines the point on the floor on which the robot must step in order to come to a complete stop, that is, where the linear momentum is eliminated Pratt et al. (2006). It is a characteristic point within the 3D-LIPM and therefore assumes that the robot has mass-less legs and that there is no acting angular momentum. Considering the dynamics within the LIPM where all the mass is located at the position of the CoM *p*_*c*_, we can formulate the equations of motion as follows:

$${\ddot{p}}_{c}\left(t\right)=\omega_{e}^{2}\left(p_{c}\left(t\right)-p_{z}\right)$$ (10)

where *ω*_*e*_ is the eigenfrequency of the pendulum itself. When modeling the multibody system as 3D-LIPM, the ZMP *p*_*z*_ is not fixed but can move inside the BoS. We assume that at *t* = 0, the robot maintains *p*_*z*_ at a constant location and *ω*_*e*_ is also modeled constant. The assumption leads to the following differential equation of the horizontal components of *p*_*c*_, as follows:

$$p_{c}\left(t\right)=\frac{1}{2}\left(p_{c_{0}}-p_{z}+\frac{{\dot{p}}_{c_{0}}}{\omega_{e}}\right)e^{\omega_{e}t}+\frac{1}{2}\left(p_{c_{0}}-p_{z}-\frac{{\dot{p}}_{c_{0}}}{\omega_{e}}\right)e^{-\omega_{e}t}+p_{z}$$ (11)

where $p_{c_{0}}=p_{c}\left(0\right)$ and ${\dot{p}}_{c_{0}}={\dot{p}}_{c}\left(0\right)$ are the initial CoM position and velocity. With a moving CoM, the linear momentum can only be removed by finding a constant value for *p*_*c*_(*t*) with *t* → *∞*. As *t* → *∞*, we obtain a divergent term in *p*_*c*_(*t*) as $e^{\omega_{e}t}\to \infty$ . To avoid a divergence of the coefficient, we apply the following condition, so the exponential is equal to zero, as seen below:

$$p_{c_{0}}+\frac{{\dot{p}}_{c_{0}}}{\omega_{e}}=p_{z}$$ (12)

Equation 12 shows that the limits of CoM position and velocity are $\underset{t\to \infty }{\lim}p_{c}=p_{z}=p_{cp}$ and $\underset{t\to \infty }{\lim}{\dot{p}}_{c}\left(t\right)=0$ with *p*_*cp*_ called the capture point. Using (Eq. 12) for any position of the CoM, we can define the CP considering the aforementioned equivalence as follows:

$$p_{cp}=p_{c}+\frac{{\dot{p}}_{c}}{\omega_{e}}$$ (13)

The CP can now be used to determine a position during locomotion that allows the robot to control the momentum acting on the CoM by stepping on a point that ensures a statically stable joint configuration. We use the CP to draw a conclusion about the dynamic stability, since the CP takes into account the unstable COM dynamics and is applied on a variety of different robot systems.

• Foot Placement Estimator (FPE): Similar to the CP, the FPE Wight et al. (2008) uses a pendulum model to calculate where the CoP should be placed with respect to the CoM so that the robot can achieve a statically balanced standing position. Although the FPE is similar to the CP, it additionally considers linear as well as angular momentum about the CoM. The total leg length *L*(*φ*) of the robot within the LIPM is calculated as follows:

$$L\left(\varphi \right)=\frac{h_{n}}{cos\left(\varphi \right)}$$ (14)

where *φ* describes the angle of a line perpendicular between the COM and the ground and the leg of the pendulum. For calculation of the FPE, we assume that the AM about the FPE is conserved during contact of the foot, as shown below:

$$H_{f1}=H_{f2}$$ (15)

*H*_*f*1_ refers to the AM before and *H*_*f*2_ to the AM after the contact of the foot. This term is extended as follows:

$$mL\left(\varphi \right)\left(v_{x}cos\left(\varphi \right)+v_{y}sin\left(\varphi \right)\right)+J_{C}\omega_{1}=\left(mL{\left(\varphi \right)}^{2}+J_{C}\right)\omega_{2}$$ (16)

where *v*_*x*_ and *v*_*y*_ are the global horizontal and vertical velocities of the CoM, respectively. The moment of inertia-weighted average angular velocity is defined as *ω*_1_ and *ω*_2_, referring to the angular velocity of the model after foot contact. The conservation of energy after foot contacts is defined as follows:

$$K2+P2=mgh_{\mathrm{peak}}$$ (17)

where *K*2 and *P*2 denote the kinetic and potential energy, respectively. The maximum CoM height which is equal to the CoM height in a LIPM is defined as *h*_*peak*_. Eq. 17 can thus be rewritten as follows:

$$\frac{1}{2}\left(J_{C}+mL{\left(\varphi \right)}^{2}\right)\omega_{2}^{2}+mgL\left(\varphi \right)cos\left(\varphi \right)=mgL\left(\varphi \right)$$ (18)

We obtain the horizontal FPE location by solving the following:

$$p_{f}=p_{c}\left(x\right)+L\left(\varphi \right)sin\left(\varphi \right)$$ (19)

where *p*_*c*_(*x*) is the global x coordinate of the CoM. We utilize the location of the FPE to describe the dynamic stability margin relative to the BoS *d*_*B*_ *F*, taking into account the specific foot position and dynamics of the movement Millard et al. (2012). We also follow this approach for the CP and the ZMP, where we also use the CP-BoS and ZMP-BoS distances *d*_*B*_ *C* and *d*_*B*_ *Z*. In general, as with the CP, the FPE allows us to make a statement about the dynamic stability of the system by also considering the AM about the CoM.

• In addition, we evaluate differences in joint angles obtained from the target trajectory and the robot’s measurement data. This allows us to determine the difference between the two trajectories from robot and simulation *d*_*R*_ *S* and identify which motors of the robot had difficulty providing the required torque given the dynamic system characteristics to follow the target trajectory. In the process, we also consider the effects on CoM position and velocity.

The given metrics are calculated for every recorded time stamp and further examined using various aggregation functions. The calculation for each time stamp is also important with respect to the CP and FPE criteria, whose conditions are based on a constant COM height and a constant leg length, respectively. Due to the complete recalculation, the criteria are re-evaluated at each time sample, which makes it possible to apply these metrics also to StS transfers, provided that they are not used as an actual control architecture but only for subsequent evaluation purposes. We determine the mean and standard deviation of a metric as well the percentage of the criteria staying inside and outside the BoS, the minima and maxima, and the norm of the underlying metrics normalized by experimental duration. We report the aggregated data as PIs of the underlying metrics.

## 3 Sit-to-Stand Motion as Optimal Control Problem

The sit-to-stand motion is obtained by solving a two-phase optimal control problem (OCP) and executed by the robot within an open-loop system: during the first phase, the robot sits on the chair. Through upper body movements only, the robot shifts its center of pressure forward toward the feet. The second phase starts when the contact forces at the chair vanish. The robot lifts itself up and assumes a predefined standing pose. We reduced the complexity of the problem by reducing the robot model to the sagittal plane, since the motion only requires movement within this plane. The reduced robot model has 10 (7 internal) DoF. The joints considered are the ankle, knee, hip, torso, head, shoulder, and elbow flexion/extension, with the left and right leg and the left and right arm lumped together. The OCP is formulated as follows. For better readability, the time dependencies are omitted:

$$\underset{q,\dot{q},\tau ,u,T_{1},T_{2}}{\min}\Psi \left[q,\dot{q},\tau ,u,T_{1},T_{2}\right]:=\int_{0}^{T_{1}+T_{2}}\phi \left(q,\dot{q},\tau ,u\right)dt$$ (20a)

$$s.t.\left\{\begin{array}{ccc} & M\left(q\right)\ddot{q}+G_{i}{\left(q,p\right)}^{T}\lambda =\tau -C\left(q,\dot{q}\right)\; & \left(20b\right)\\ & \dot{\tau }=u\; & \left(20c\right)\\ & g_{i}\left(q,p\right)=0\; & \left(20d\right)\\ & {\hat{h}}_{i}\left(q,\dot{q},\tau ,u,T_{1},T_{2}\right)=0\; & \left(20e\right)\\ & h_{i}\left(q,\dot{q},\tau ,u,T_{1},T_{2}\right)\geq 0,\;i=1,2\; & \left(20f\right)\\ & \underline{q}\leq q\leq \bar{q},\; & \left(20g\right)\\ & \underline{\dot{q}}\leq \dot{q}\leq \bar{\dot{q}},\; & \left(20h\right)\\ & \underline{\tau }\leq \tau \leq \bar{\tau },\; & \left(20i\right)\\ & \underline{u}\leq u\leq \bar{u}\; & \left(20j\right) \end{array}\right.$$ (20)

with *q*, $\dot{q}$ , and $\ddot{q}$ being the joint positions, velocities, and accelerations, respectively. *T*_1_ and *T*_2_ are free variables describing the phase duration for the contact phase with the chair (*T*_1_) and the lifting phase to the standing position (*T*_2_), which are also determined by optimization. These times result implicitly from the optimized motions as the times when the force between the robot and the chair reaches zero and when the robot is reaching standing position. The controls *u* are the derivatives of the joint torques *τ*, also indicated by (Eq. 20c). The objective function (Eq. 20a) consists of a Lagrange term over the duration of the whole motion. The function *ϕ* is described in detail in Section 3.2.

The equation of motion of the robot as a constrained multibody system is denoted by (Eq. 20b) with mass matrix *M*, constraint jacobian *G*_*i*_, and unknown force variables *λ*. The centrifugal, gravitational, and Coriolis forces are summarized by *C*. During the first phase, three contacts are applied on the model: one at the back and front of the robot’s foot and one at the thigh segment representing the contact with the chair. The contact to the chair is lost at the transition to the second phase. The foot contacts remain. The parameters p specify the chair height $p^{\bar{GC}}\in \left\{49.8\;cm,47.7\;cm,45.7\;cm,43.6\;cm,41.5\;cm\right\}$ and the distance between the chair and the ankle origin of the robot $p^{\bar{CA}}\in \left\{10.4\;cm,12.5\;cm,14.5\;cm,16.6\;cm\right\}$ (Table 1), varying either $p^{\bar{GC}}$ or $p^{\bar{CA}}$ for each calculation. The constraints (Eq. 20d) match the contact points at the thigh and the feet to the respective specified locations.

The constraints (Eq. 20e) and (Eq. 20f) define key characteristics of the motions that are described in the following subsection 3.1. Box constraints on the states and controls are denoted by (Eqs 20g–20j). The limits for the joint angles, velocities, and torques were adopted from the robot’s manual. The torque limit of the hip joint had to be adjusted in order to successfully execute the generated motions on the real robot. The direct multiple shooting method is used to discretize the OCP, and the resulting nonlinear program is solved using the sequential quadratic programming and active-set method provided by MUSCOD-II Leineweber et al. (2003). RBDL is used for calculating the rigid multibody dynamics.

### 3.1 Constraints

The applied constraints (Eq. 20e) and (Eq. 20f) can be distinguished into constraints that are to be satisfied at a particular time, at the beginning (S) or at the end (E) of a phase and constraints that are to be satisfied during the entire phase (I). All constraints applied at (S) and (E) of a phase are equality constraints [see (Eq. 20e)], while all constraints defined over the entire phase duration (I) are inequality constraints (see Eq. 20f) in this OCP:

• Phase 1—Starting Conditions (S): The upper body is fixed in a predefined position. Otherwise, the OCP calculates an upper body that is already leaning forward with the arm extended, as this reduces the effort considerably. The position of the lower body is determined by the contact points specified by $p^{\bar{GC}}$ and $p^{\bar{CF}}$ . At the beginning of the motion, the robot sits at rest. Therefore, the joint velocities are set to zero.
• Phase 1—Interior Conditions (I); also included in Phase 2—(I): The normal contact forces acting at the feet should be positive, that is, the feet are lying on top of the ground and are not fixated on the ground. Furthermore, the horizontal contact forces acting at the feet and at the chair contact points should stay within the respective friction cones, so that no sliding of the segments occurs during the motion. To prevent the arm from colliding with the chair, a constraint placing it in the front of the chair during the whole motion was included as well.
• Phase 2—Starting Conditions (S); corresponds to Phase 1—(E): The normal force acting at the chair contact vanishes. The contact to the chair is lost, and the thigh is leaving the chair.
• Phase 2—Additional Interior Conditions (I): The position of the virtual thigh contact in space should remain above the contact point of the chair to avoid collisions when standing up. The CoP is limited to a maximum deviation from the ankle of (−1.5 cm; 4 cm). When the CoP was allowed to reach the boundaries of the support polygon of the real robot, this resulted in motions that were not successfully executed on the real robot, probably due to a mismatch of the model used in the optimization caused by, for example, neglected motor dynamics.
• Phase 2—Ending Conditions (E): The robot should adopt a pre-defined end position, the general standing posture proposed by the manufacturer, and it should be at rest. Thus, the joint velocities and accelerations should be zero.

### 3.2 Objective Function

The cost function (Eq. 20a) combines three common objectives for motion generation: minimization of joint torques, minimization of joint torque change, and minimization of mechanical work:

$$\phi \left(q,\dot{q},\tau ,u\right)=\;\tau^{T}W_{t}\tau +u^{T}W_{u}u+|\tau^{T}W_{m}\dot{q}|$$ (21)

with *W*_*t*_, *W*_*u*_, and *W*_*m*_ diagonal matrices specifying a joint-dependent weighting of the corresponding terms. They were determined heuristically and in accordance to the size of the quantities, giving the joints with expected small torque and torque change values, such as the head and the arms, a higher scaling factor. The minimization of torques reduces the amount of torque needed to perform the motion, which is important, but also results in very dynamic movements that use a lot of swinging of the limbs to generate the required torques efficiently (resulting in an exploitation of the motion dynamics). We balance this by minimizing the mechanical work, which yields slow and controlled movements. The minimization of torque change reduces redundancies in the OCP and also avoids high changes in the torque that the motors may not be able to realize, thus making the solution feasible on the robot.

## 4 Results

**FIGURE 8:**
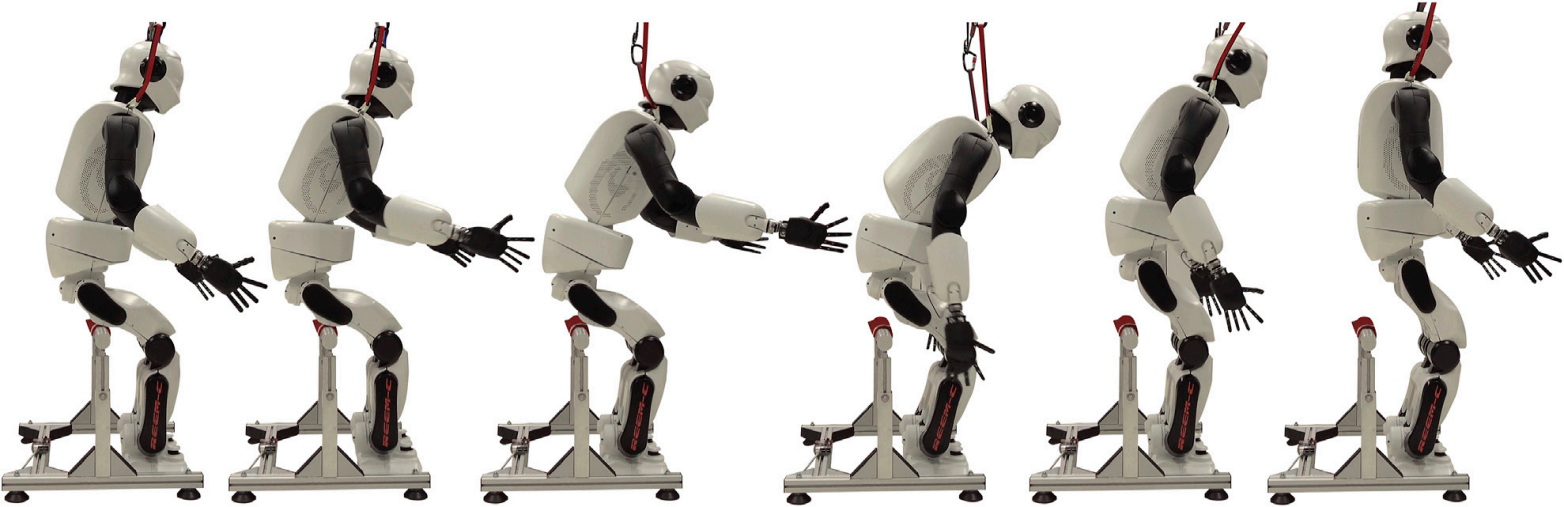
Image sequence shows a dynamic sit-to-stand transition of the second protocol *P*2 at 40% difficulty setting performed by the humanoid robot REEM-C. The robot reaches the required momentum for standing up by raising and instantaneously lowering both arms. The contact is released during the lowering motion of both arms, after which the robot lifts its upper body by means of the hip motors.

**FIGURE 9:**
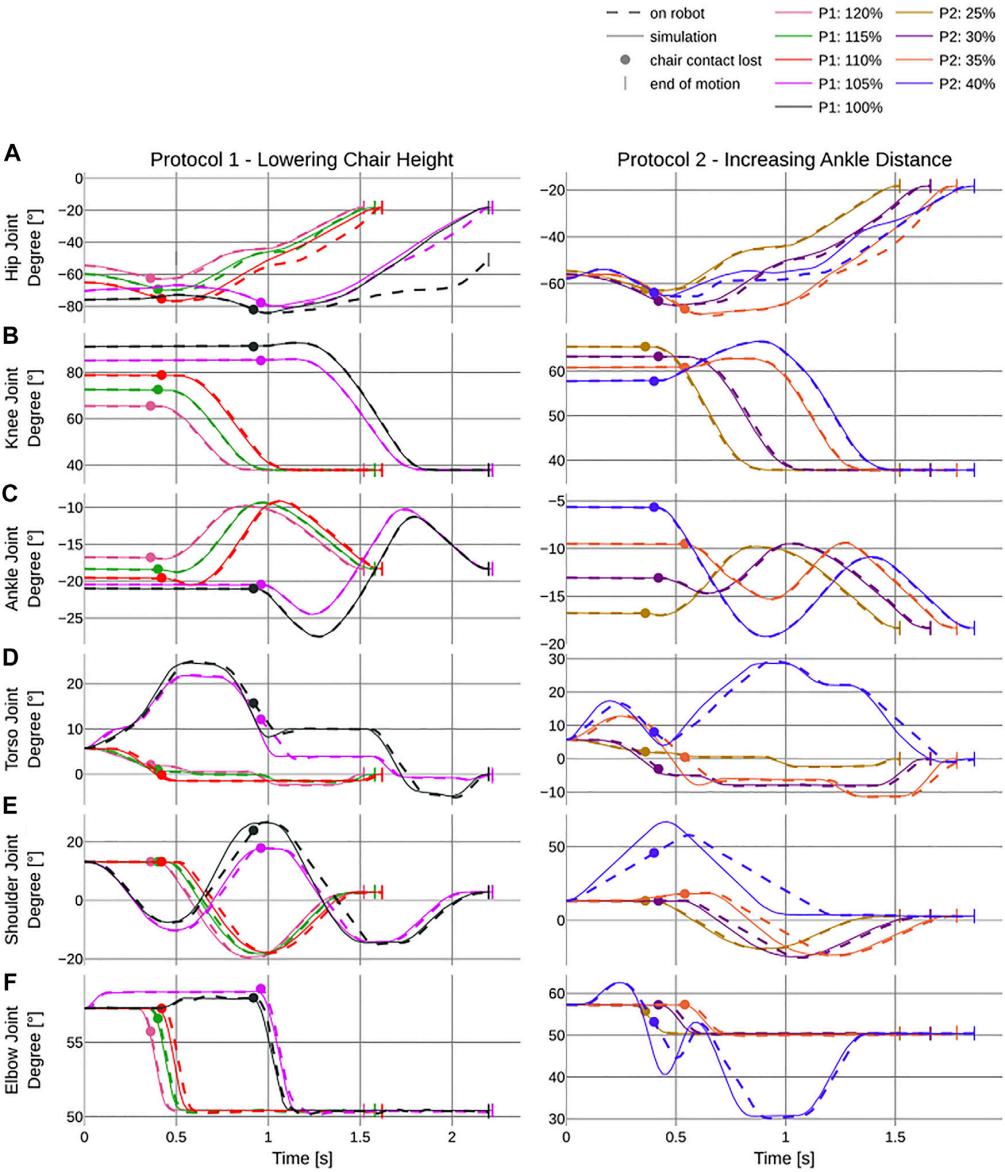
Deviation of simulated (solid line) and actual (dashed line) joint trajectories over the duration of the different trials, in seconds. The left column shows the corresponding trajectories to the first protocol which incrementally lowers the chair height and the second protocol where distance between the center of the chair and the center of the ankle joint is increased. All trials are in % of knee height (see Table 1). **(A–F)** report the different joint trajectory profiles of the hip, knee, ankle, torso, shoulder, and elbow joint, respectively, for the flexion and extension of the corresponding limbs. The dot indicates the time of breaking contact with the chair and the pipe symbols indicate the end of each trial.

The OCP (Section 3) generated eight different StS trajectories, each of which the robot could execute for three consecutive times (Figure 8). By analyzing the difference between the simulated and actual joint trajectories, we observe that the robot has difficulties maintaining the target trajectory in its hip joints breaking contact with the chair between the first and the last motion of *P*1 [0.98 (1.3) vs. 9.51 (13.81) ° Figure 9A]. This is reflected especially by the fact that the robot fails to reach the target setting within the given time. For the ankle, elbow, and torso joints, the robot was able to stick closely to the target trajectory (Figures 8B–D). The shoulder joint diverges further from the target trajectory the more difficult the trial [0.38 (0.52) vs. 1.98 (2.49)°, Figure 8E], whereas the elbow closely matches (Figure 9F). Protocol *P*2 confirms the trend of higher deviation for more complex trials, as the hip joint diverges further but not to the same extent as for *P*1 ]0.42 (0.44) vs. 2.43 (2.43) °, Figure 9A]. For *P*2, the elbow joint closely matches again, but we identify higher deviations in the torso joint for all trials but especially among the last trial in *P*1 [0.72 (0.86) °] and *P*2 [1.62 (1.36) °, Figures 9B–D]. We identify peaks for the shoulder trajectory and overall a higher deviation for the more difficult trials in *P*2 than in *P*1 (6.72 (6.95) vs. 1.98 (2.49) °, Figure 9E]. The same applies to the elbow joint for *P*2 at $|d_{RS}^{\mathrm{elbow}}{|}_{2}\times 1/t$ being 12.97 which had almost no deviation in *P*1 with 0.97 and therefore diverges a lot for the most difficult trial *P*2 at 40% [1.35 (2.12)°, Figure 9F]. For *P*1 and *P*2, the knee joints for both simulation and robot match closely (see Section 6).

**FIGURE 10:**
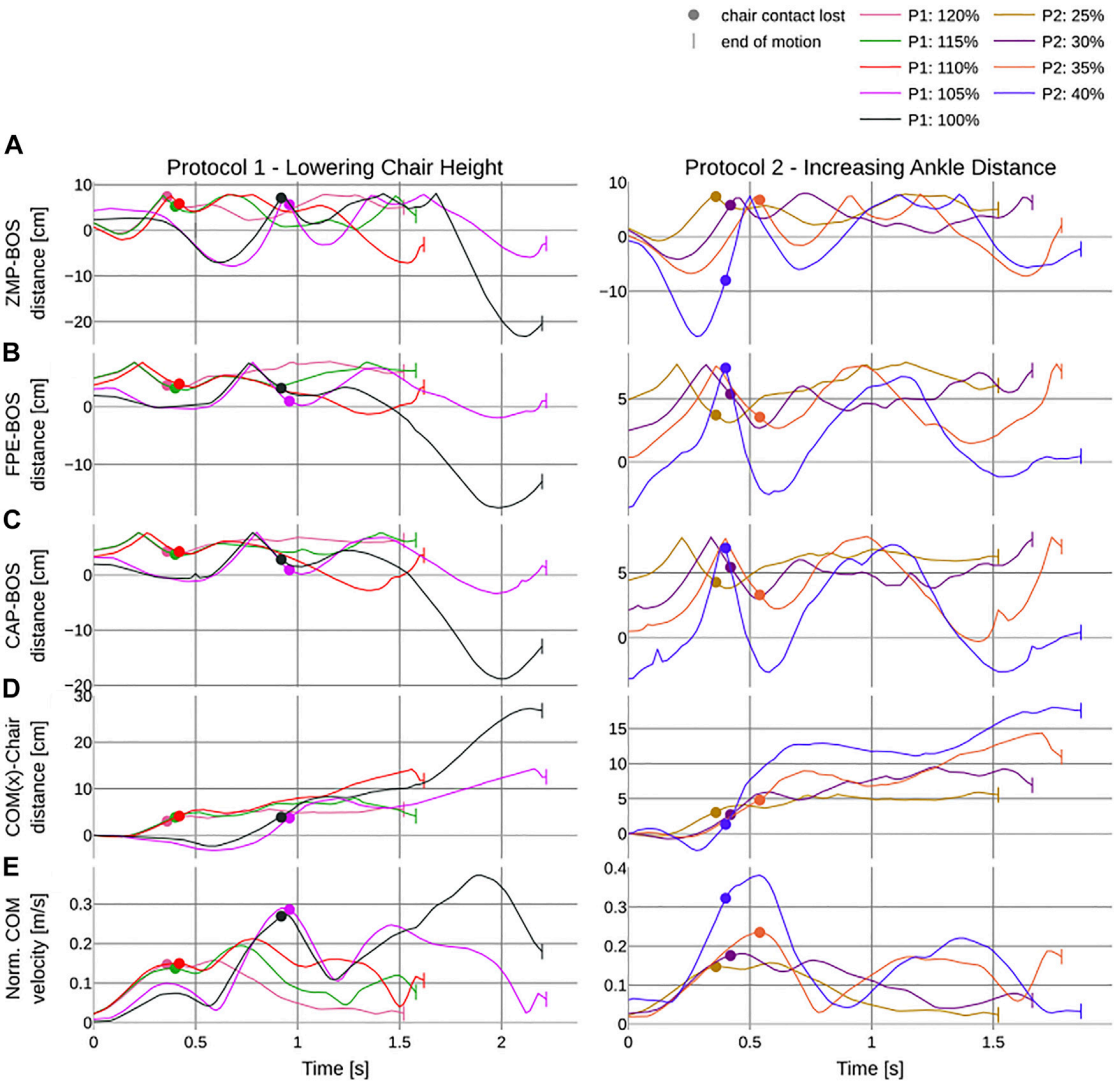
Results of the calculated PI over the duration of the different trials in seconds. The left column shows the corresponding trajectories to the first protocol which incrementally lowers the chair height and the second protocol where distance between the center of the chair and the center of the ankle joint is increased. All trials are in % of knee height (see Table 1). **(A–C)** report the distance of the point-based metrics to the base of support: ZMP–, FPE–, and CP–BoS distances in cm, respectively. **(D)** reports the trajectory of the x component of the CoM. **(E)** reports the L2 norm of the CoM velocity. The dot indicates the time of breaking contact with the chair and the pipe symbols indicate the end of each trial.

The point-based metrics report decreasing stability with increasing difficulty. For *P*1, break of contact only happens while the BOS-ZMP distance *d*_*BZ*_ is positive (Figure 10A). For the two easier trials in *P*1 *d*_*BZ*_, there is a slight divergence into the negative (6.49 and 7.5% of the time, respectively), whereas for the two more difficult trials, a major divergence into the negative right before breaking contact can be observed. The second most difficult trial is longer still in the negative ranges (43.8 vs. 36.9%). The L2 norm |*d*_*BZ*_|_2_ × 1/*t* indicates a major divergence with 319 vs. 213 for the two most difficult trials *P*1, 105 and 100%. The FPE-BOS distances *d*_*BF*_ all start in the positive, except for *P*2 at 40% (Figure 10B). The two more difficult trials in *P*1 *d*_*BF*_ almost become negative right before break of contact. For all trials in *P*1, a positive peak can be identified right before breaking contact. For *P*1, *d*_*BF*_ resides outside the BoS with increasing regularity, as indicated by the percentage ranging from 100% inside to only 61.3% for the most difficult trial. For *P*1 this is only the case for the last and most difficult trial. *P*2 40% is also the only motion with negative *d*_*BF*_ at −3.62 cm for the initial configuration. In contrast to all other trials, contact is broken right at the peak of *d*_*BF*_ at 7.44 cm. The CP follows the trend of the FPE with *d*_*BF*_ and *d*_*BC*_ being almost identical for all the trials (Figure 10C) (see Section 6).

The CoM location about the x-axis which equals zero at the contact point on the chair becomes more negative the more difficult the initial configuration (Figure 10D). For *P*1 at 105% and 100% and for *P*2 at 40%, min (*CoM*_*x*_) is reported with −3.25, −2.36, and −2.38 cm, respectively. The normalized CoM velocity for all trials except *P*2 40% loses contact at the first peak velocity (Figure 10E). After each loss of contact, a decline in velocity can be observed, which becomes more significant as the level of difficulty is increased. The peak velocity also increases the more difficult the initial configuration, except for *P*1 100% which peaks right before the end of the experiment (see Section 6).

**FIGURE 11:**
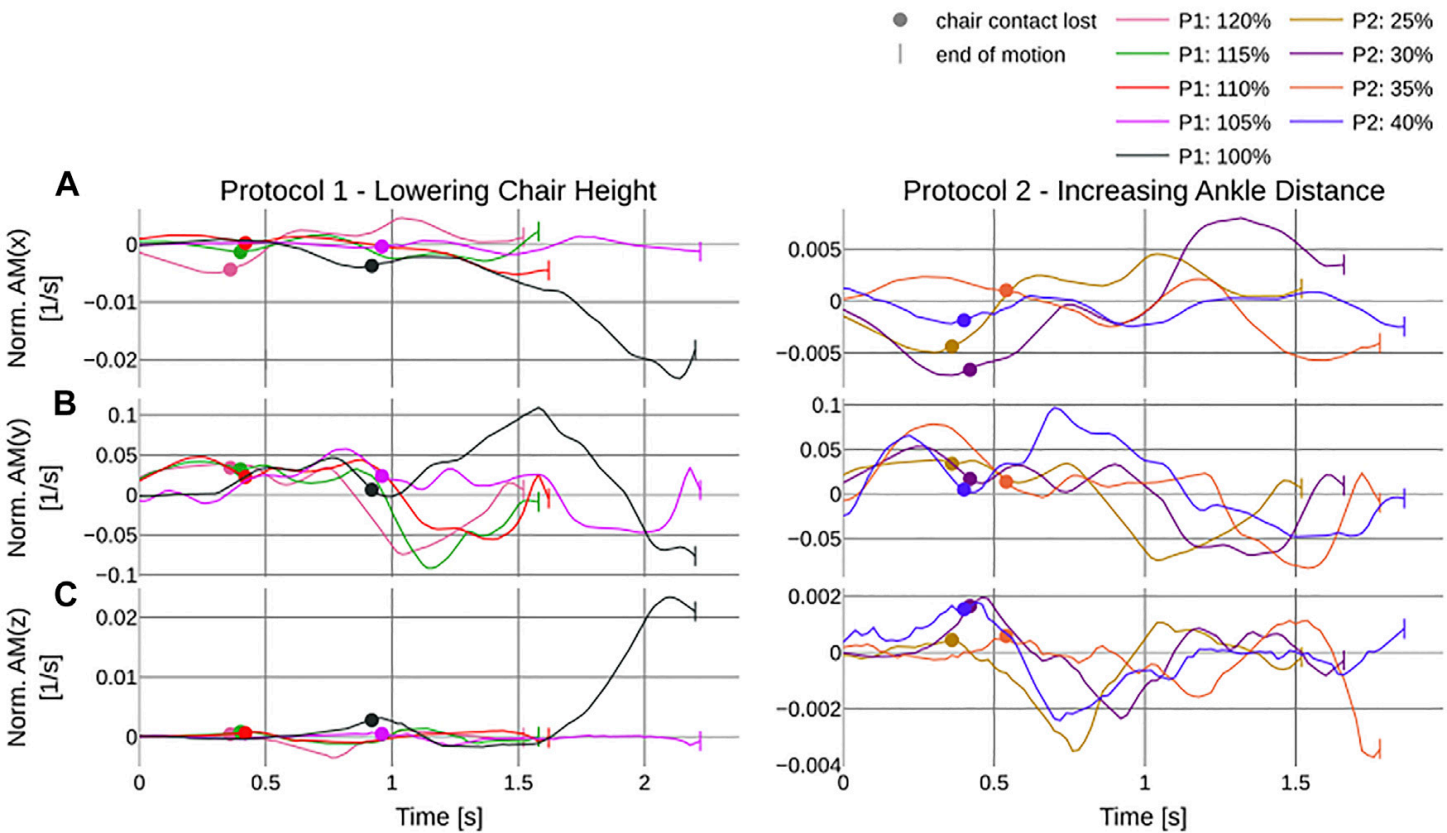
Results of the angular momentum about the CoM normalized by *ml* ^2^ where *m* is the mass and *l* the leg length of the REEM-C over the duration of the different trials in seconds. The left column shows the corresponding trajectories to the first protocol which incrementally lowers the chair height and the second protocol where distance between the center of the chair and the center of the ankle joint is increased. All trials are in % of knee height (see Table 1). **(A–C)** report the normalized angular momentum about the x, y, and z axis, respectively. The dot indicates the time of breaking contact with the chair and the pipe symbols indicate the end of each trial.

The normalized angular momentum around the CoM for *AM*_*y*_ shows higher values even for the most simple trial of 120% in *P*1 [0.0331 (0.0178) 1/s] compared to 100% *P*1 [0.0386 (0.0306) 1/s] and 40% *P*2 [0.0379 (0.0234) 1/s]. For each of the trials, angular momentum is accumulated to a different extent before the contact is broken. In all cases, the contact breaks at the time when the previously accumulated *AM*_*y*_ is utilized (Figure 11B). The 100% trial reaches the highest peak with 0.1091 1/s approximately 0.6 s after breaking contact. For all trials, *AM*_*y*_ stays within a boundary of (−0.0916, 0.1091) 1/s. *AM*_*x*_ (Figure 11A) and *AM*_*z*_ (Figure 11C) stay within very small boundaries (see Section 6).

The evaluated force data measured by the force sensors of the robot and the chair were not as concise. For protocol *P*1, the impact forces of the robotic feet were lower (between 700 and 1005 N) than for protocol *P*2 (between 800 and 1050 N), with the easiest trial *P*1 120% being comparatively high at 1005 N. The highest impact force was measured for the most difficult trial, *P*2 40% with 1048 N. On the chair, a maximum load of 901 N was measured at the second most difficult trial in protocol *P*1 at 105%, compared to the most difficult trial 100% with 766 N. For protocol *P*2, the impact force increases consistently and ranges from 623 N for 25% to the highest measured force on the chair at 854 N for 40% (see Section 6).

The duration of the individual trials increased continuously with increasing difficulty. Thus, for *P*1, the time needed to stand up increased from 1.54 up to 2.22 s and for *P*2, from 1.54 s up to 1.87 s (see Section 1).

## 5 Discussion

In order for a robot to move out of the laboratory and into a built-for-human environment, humanoid robots must be able to perform complex motion tasks. Furthermore, such motion sequences are also ideal benchmarks for measuring the actual capabilities of a mechanical system. In this work, we applied optimal control to generate different StS trajectories. The parameter selection for the OCP was based on a unified protocol we developed to determine the performance of the lower extremities. The difficulty of the initial configuration was methodically increased until the robot could no longer perform the motion. Based on the data obtained, various PIs were calculated and analyzed based on well-known metrics.

The results support optimal control as a tool for generating humanlike motion. The OCP was able to generate very dynamic joint trajectories which temporarily put the ground-projected CoM outside the BoS, leaving the robot in a statically unstable state. Due to the momentum acting on the mechanical system, the robot still remained dynamically stable. For a position-controlled and not compliant robot, this in itself is a great achievement. Nonetheless, there is further room for improvement in the formulation of the OCP as the foot and sitting contact are formulated as rigid contacts and the gearbox of the motors is not modeled.

When we compare the results of the point-based metrics with the performance of young and elderly humans, we identify that REEM-C provides almost identical performance, thus proving to be a good indicator for determining human likeness. While executing the OCP trajectories, REEM-C compares to younger adults showing a *d*_*BF*_ at [5.7 (1.4) cm] and an StS duration of [1.7 (0.2) s] Sloot et al. (2020) with *d*_*BF*_ [5.03 (1.31) cm] at 1.66 s StS duration for similar experimental conditions (protocol *P*2 at 30%). When comparing the overall motions generated, the OCP trajectories executed by REEM-C are in general more comparable to the performance of younger adults from the aforementioned publication, as they are less conservative. We therefore performed the same protocol for ten human subjects in their mid-20s. While *d*_*BF*_ was in a similar range for the difficulty levels performed by the robot, it was possible for the human subjects to master more difficult configurations. As the robot only managed a chair decrease of 100% of its knee height in *P*1, the human subjects were generally able to get up from chairs up to 45% of their knee height and up to 75% ankle distance for *P*2 and were thus able to realize a significantly higher degree of difficulty. At this point, it should be said that the optimization problem for *P*1 was able to calculate solutions down to 85% knee height and up to 45% ankle distance for *P*2 within the simulation. To perform such difficulty settings on the real robot, closed-loop feedback control is required to prevent an overall oscillation of the mechanical system. Nevertheless, the motions executed by the robot are the first dynamic stand-up motions successfully executed by REEM-C. The only previously existing stand-up motion supplied with the robot, with a total duration of 10 seconds, serves as a reference, whereby T1 lasted 4.98 s until the robot shifted the COP forward by means of its arms and completely stood up within an additional 5.02 s in T2.

The version of the REEM-C robot used, which was developed specifically for use as a standard platform within the EUROBENCH project, was equipped with more powerful knee motors targeted specifically for scenarios such as ascending stairs, carrying objects, or walking over various obstacles. Our results show that these new motors do not have any difficulties following the trajectories. Most of the problems arise within the hip and torso motors, which have not been updated. Thus, for the OCP to generate motions that execute successfully on the robot, the maximum allowed hip-torques (according to the manual) had to be further corrected downward. For more difficult trials, the robot is still unable to move the hip joints against the downward momentum caused by gravity when the robot tries to reach an upright posture in an impulsive motion after leaving contact. This is confirmed by the results of both protocols, which are conclusive based on the comparison of the actual and target trajectories and are thus able to highlight deficits in the required motor strength. Especially for *P*1 at 100%, this becomes evident as the robot reaches the maximum applicable hip torques and cannot accurately follow the given target trajectory. Protocol *P*1 with lower chair height in general evaluates the hip motors of the robot since the largest deviations are present here, while protocol *P*2 generates larger deviations in the torso joint due to the ankle position being shifted forward. An unforeseen result which can be observed is also that the joints within the arms have problems following the trajectory, which can only be related to the large impulse within the kinematic chain. No further load on the arms is present, and for more classical motion sequences, no deviation has been detected to date. The separation of the experiment into two different protocols thus proves to be useful for analyzing different deficiencies within the system. Although REEM-C was able to handle the provided motions very well, at this point, it must be mentioned that the detected shortcomings will not be limited to StS scenarios, but will affect all scenarios with vertical CoM displacement, such as pick and place or carrying tasks.

While the point-based metrics ensure good comparability within the benchmark, the normalized angular momentum was able to make a statement across experiments for its stability and human likeness. Similar to humans, *AM*_*x*_ *AM*_*y*_ and *AM*_*z*_ are also close to zero based on the OCP trajectories performed by the robot. In a previous publication dealing with stability during bipedal walking, stability regions were identified within which the robot can operate without risking a loss of balance Aller et al. (2021b). The data evaluated in this publication are within the same region and do not violate its boundaries, which is why the movements could ultimately be executed.

## 6 Conclusion

StS motions, or more specifically, StS motions obtained by means of optimal control, have proven to be an effective approach for evaluating the whole-body motion performance of a bipedal multibody system. The robot is able to stand up from a minimum chair height corresponding to 100% of its knee height and to place the ankles at 40% of its knee height away from the sitting contact. Thus, for the respective protocols *P*1 and *P*2, REEM-C achieves a StS score of 100 and 40, corresponding to its determined knee height. The metrics applied were able to provide an indication of the robot’s performance. Thus, deficiencies in the mechanical system were uncovered, and the performance in relation to other motions could be evaluated. The underlying framework is implemented on standardized formats. The results are normalized and allow a robot-independent comparability with the results reported based on the standardized protocol. Future research will therefore be based on the same unified framework to analyze further motion tasks and determine the capability of humanoid robots by abstracting the requirements of specific applications in different operational domains.

## Data Availability

The datasets presented in this study can be found in online repositories. The names of the repository/repositories and accession number(s) can be found below: https://github.com/flxalr/sts-optimization-reemc/.
