# Supplementary material for: Optimization of Dynamic Sit-to-Stand Trajectories to Assess Whole-Body Motion Performance of the Humanoid Robot REEM-C
Source: Front Robot AI. 2022 Jun 28;9:898696. doi: 10.3389/frobt.2022.898696 (PMC9273909; doi:10.3389/frobt.2022.898696)
Supplement: Supplementary file 1 [file DataSheet1.PDF]

Supplementary Material

1 CUBIC SMOOTHING SPLINE FIT

For obtaining missing data (joint acceleration, base link velocity and acceleration) we apply a cubic smoothing spline fit the source data De Boor (1978). The smoothing spline minimizes the term

$$p \sum_{j=i}^{n} w_j |y_j - f(x_j)|^2 + (1-p) \int \lambda(t) |D^2 f(t)|^2 dt \tag{S1}$$

where the first and second term are for the error and roughness measure, respectively. The second derivative of f is denoted as $D^2 f$. We apply a smoothing parameter p of 0.99.

2 EXPERIMENTAL DATA

Table S1. Differences in joint angles. The table compares the differences of the actuated joints during the StS-transitions among all trials for the two different protocols $P1$-lower chair height and $P2$-increased ankle distance. For each joint the avg. [std] and the L2 norm of the difference in degrees between the actual and simulated data are reported.

	Trial	Hip Joint		Knee Joint		Ankle Joint	
Protocol 1	115%	0.98 [1.3]	9.14	0.07 [0.11]	0.72	0.04 [0.03]	0.29
	110%	3.41 [3.24]	25.94	0.28 [0.43]	2.83	0.15 [0.14]	1.14
	105%	2.12 [2.66]	16.14	0.01 [0.01]	0.07	0.01 [0.01]	0.05
	100%	9.51 [13.81]	78.69	0.04 [0.06]	0.34	0.02 [0.02]	0.16
Protocol 2	25%	0.42 [0.44]	3.44	0.13 [0.22]	1.44	0.08 [0.07]	0.57
	30%	0.77 [0.62]	5.46	0.19 [0.33]	2.1	0.12 [0.1]	0.83
	35%	1.51 [1.97]	13.14	0.1 [0.17]	1.07	0.07 [0.06]	0.51
	40%	2.43 [2.43]	17.73	0.06 [0.08]	0.54	0.05 [0.04]	0.33

		Torso Joint		Shoulder Joint		Elbow Joint	
Protocol 1	115%	0.11 [0.09]	0.8	0.09 [0.13]	0.88	0.09, [0.13]	0.88
	110%	0.19 [0.24]	1.68	0.83 [0.88]	6.67	0.15, [0.34]	2.03
	105%	0.54 [0.93]	5.1	0.75 [0.73]	4.96	0.1, [0.14]	0.8
	100%	0.72 [0.86]	5.31	1.98 [2.49]	15.06	0.11, [0.18]	0.97
Protocol 2	25%	0.11 [0.06]	0.71	0.38 [0.52]	3.66	0.07 [0.08]	0.62
	30%	0.4 [0.45]	3.34	1.6 [1.93]	13.78	0.15 [0.34]	2.03
	35%	1.11 [1.49]	9.84	2.01 [2.91]	18.71	0.15 [0.24]	1.48
	40%	1.62 [1.36]	10.93	6.72 [6.95]	49.87	1.35 [2.12]	12.97

Table S2. Results based on the point based metrics. The table shows the distance from the location of the corresponding point based metric to the Base of Support (BoS) among all trials for the two different protocols $P1$-lower chair height and $P2$-increased ankle distance. For each metric and trial the aggregate functions report the avg. [std], minima, maxima, percentage [inside, outside] of the BoS and the integrated values normalized by experimental duration.

		Trial	Average	Min	Max	Percentage	Integral
**ZMP**	**Protocol 1**	115%	3.33 [2.59]	-0.81	7.8	[92.5, 7.5]	170.17
		110%	2.13 [4.49]	-7.16	7.9	[67.07, 32.93]	219.38
		105%	0.68 [4.73]	-7.88	7.8	[56.25, 43.75]	213.01
		100%	-1.43 [8.78]	-23.23	8.05	[63.06, 36.94]	318.68
	**Protocol 2**	25%	4.47 [2.43]	-0.71	7.85	[93.51, 6.49]	224.88
		30%	2.79 [3.15]	-4.14	7.97	[83.33, 16.67]	183.34
		35%	0.12 [4.41]	-7.21	7.84	[48.89, 51.11]	187.42
		40%	-1.75 [6.73]	-18.28	7.79	[39.36, 60.64]	279.94

		Trial	Average	Min	Max	Percentage	Integral
**FPE**	**Protocol 1**	115%	5.31 [1.26]	2.92	7.74	[100.0, 0.0]	263.46
		110%	3.03 [2.41]	-1.28	7.67	[82.93, 17.07]	163.56
		105%	1.96 [2.54]	-1.91	7.7	[70.54, 29.46]	120.97
		100%	-2.39 [7.37]	-17.48	7.54	[61.26, 38.74]	260.06
	**Protocol 2**	25%	6.02 [1.25]	3.13	7.9	[100.0, 0.0]	297.9
		30%	5.03 [1.31]	2.48	7.7	[100.0, 0.0]	251.04
		35%	4.12 [2.06]	0.36	7.75	[100.0, 0.0]	205.61
		40%	1.49 [2.96]	-3.62	7.44	[64.89, 35.11]	123.26

		Trial	Average	Min	Max	Percentage	Integral
**CP**	**Protocol 1**	115%	5.2 [1.11]	3.55	7.64	[100.0, 0.0]	258.09
		110%	2.94 [2.97]	-2.78	7.58	[78.05, 21.95]	182.11
		105%	1.74 [2.96]	-3.32	7.7	[67.86, 32.14]	135.25
		100%	-2.15 [7.63]	-18.87	7.65	[57.66, 42.34]	264.79
	**Protocol 2**	25%	5.83 [0.85]	3.81	7.68	[100.0, 0.0]	288.53
		30%	4.75 [1.29]	2.13	7.72	[100.0, 0.0]	237.14
		35%	3.72 [2.5]	-0.28	7.78	[95.56, 4.44]	186.12
		40%	1.34 [3.34]	-3.17	7.15	[54.26, 45.74]	140.79

Table S3. Results of the COMx location and the normalized COM velocity. The two table show the results of the calculated metrics for COM(x) location relative to the sitting contact and the normalized COM velocity among all trials for the two different protocols $P1$-lower chair height and $P2$-increased ankle distance. For each metric and trial the aggregate functions report (if applicable) the avg. [std], minima, maxima, percentage [inside, outside] of the BoS and the integrated values normalized by experimental duration.

		Trial	Average	Min	Max	Percentage	Integral
COM(x) Location	Protocol 1	115%	4.54 [2.48]	-0.38	7.96	[88.75, 11.25]	227.29
		110%	6.51 [4.34]	-0.34	14.18	[89.02, 10.98]	322.84
		105%	4.42 [5.46]	-3.25	14.29	[66.96, 33.04]	219.29
		100%	8.08 [9.17]	-2.36	27.27	[69.37, 30.63]	398.29
	Protocol 2	25%	3.82 [1.96]	-0.38	5.92	[89.61, 10.39]	189.26
		30%	5.42 [3.46]	-0.74	9.55	[83.33, 16.67]	271.6
		35%	6.99 [4.65]	-0.5	14.36	[90.0, 10.0]	349.22
		40%	9.95 [6.31]	-2.38	18.03	[89.36, 10.64]	494.44

		Trial	Average	Min	Max	Percentage	Integral
Norm. COM Velocity	Protocol 1	115%	0.11 [0.04]	0.02	0.19	[100.0, 0.0]	5.48
		110%	0.13 [0.05]	0.02	0.21	[100.0, 0.0]	6.68
		105%	0.14 [0.08]	0.01	0.29	[100.0, 0.0]	7.14
		100%	0.18 [0.11]	0	0.37	[100.0, 0.0]	8.91
	Protocol 2	25%	0.08 [0.05]	0.02	0.16	[100.0, 0.0]	4.01
		30%	0.1 [0.05]	0.03	0.18	[100.0, 0.0]	5.07
		35%	0.13 [0.06]	0.02	0.24	[100.0, 0.0]	6.35
		40%	0.15 [0.1]	0.03	0.38	[100.0, 0.0]	7.63

Table S4. Minimum and maximum forces acting on the robot and chair. The table shows the z-forces in Nm measured by chair and force sensors in the robotic feet for the various difficulty settings among both trials.

		Protocol 1				Protocol 2			
		**115%**	**110%**	**105%**	**100%**	**25%**	**30%**	**35%**	**40%**
Robot	Min	-123.8	-112.4	-136.6	-168.4	-183.4	-173.4	-163.8	-129
	Max	1012	798.2	773.2	703.2	1005.6	956.2	818.2	1048.2
Chair	Min	0.32	0.1	-1.19	-3.34	0.52	0.84	0	-28.18
	Max	659.61	659.56	901.08	766.08	623.81	662.91	678.95	854.18

Table S5. Results based on the Angular Momentum (AM) metrics. The table reports the experimental results for the AM normalized by $m \times l^2$ where m is the mass and l the total leg length of the robot among all trials for the two different protocols $P1$-lower chair height and $P2$-increased ankle distance. For each metric and trial the aggregate functions report the avg. [std], minima, maxima, percentage [positive, negative] of the BoS and the integrated values normalized by experimental duration.

		Trial	Average	Min	Max	Percentage	Integral
Norm. AM(x)	Protocol 1	115%	0.0013 [0.0008]	-0.0029	0.0022	[38.75, 61.25]	0.0632
		110%	0.0017 [0.0015]	-0.0052	0.0017	[56.0976, 43.9024]	0.0851
		105%	0.0005 [0.0005]	-0.0018	0.0014	[47.3214, 52.6786]	0.0271
		100%	0.0061 [0.0069]	-0.0233	0.0009	[20.7207, 79.2793]	0.3013
	Protocol 2	25%	0.0025 [0.0014]	-0.0049	0.0045	[64.9351, 35.0649]	0.1228
		30%	0.0043 [0.0025]	-0.0071	0.008	[36.9048, 63.0952]	0.2148
		35%	0.0022 [0.0016]	-0.0057	0.0024	[50.0, 50.0]	0.1083
		40%	0.001 [0.0008]	-0.0025	0.0012	[40.4255, 59.5745]	0.0483

		Trial	Average	Min	Max	Percentage	Integral
Norm. AM(y)	Protocol 1	115%	0.0364 [0.0208]	-0.0916	0.0423	[61.25, 38.75]	1.8246
		110%	0.0339 [0.0124]	-0.0558	0.0477	[67.0732, 32.9268]	1.6903
		105%	0.0243 [0.0154]	-0.0469	0.0579	[67.8571, 32.1429]	1.2193
		100%	0.0386 [0.0306]	-0.0765	0.1091	[80.1802, 19.8198]	1.9105
	Protocol 2	25%	0.0331 [0.0178]	-0.0744	0.039	[63.6364, 36.3636]	1.6467
		30%	0.0325 [0.0197]	-0.0685	0.0533	[69.0476, 30.9524]	1.6375
		35%	0.0333 [0.0273]	-0.0833	0.0784	[67.7778, 32.2222]	1.6726
		40%	0.0379 [0.0234]	-0.0481	0.097	[57.4468, 42.5532]	1.8912

		Trial	Average	Min	Max	Percentage	Integral
Norm. AM(z)	Protocol 1	115%	0.0006 [0.0004]	-0.0013	0.0014	[46.25, 53.75]	0.0326
		110%	0.0005 [0.0003]	-0.001	0.001	[63.4146, 36.5854]	0.0237
		105%	0.0003 [0.0003]	-0.0015	0.0007	[41.0714, 58.9286]	0.0171
		100%	0.0041 [0.0068]	-0.0017	0.0234	[59.4595, 40.5405]	0.201
	Protocol 2	25%	0.0008 [0.0009]	-0.0035	0.0011	[45.4545, 54.5455]	0.0398
		30%	0.0007 [0.0006]	-0.0024	0.002	[48.8095, 51.1905]	0.0347
		35%	0.0006 [0.0008]	-0.0037	0.0011	[41.1111, 58.8889]	0.0311
		40%	0.0008 [0.0006]	-0.0024	0.0018	[52.1277, 47.8723]	0.0379

REFERENCES

De Boor, C. (1978). *A practical guide to splines*, vol. 27 (springer-verlag New York)
